# Analyzing the Effect of People Utilizing Mobile Technology to Make Banking Services More Accessible

**DOI:** 10.3389/fpubh.2022.879342

**Published:** 2022-04-29

**Authors:** Jiale Zhu, Manyi Wang

**Affiliations:** Capital University of Economics and Business, Beijing, China

**Keywords:** mobile technology, banking, financial service, transaction convenience, mobile banking

## Abstract

Many firms in the modern world utilize m-banking systems to communicate with their consumers. The word m-banking refers to a widespread method of providing financial services and localization to customers. Since m-banking is important to both banks and users, it has been included in numerous literary works. As a result, embracing financial services *via* the m-banking platform is critical. This article's technique is mostly descriptive research that investigates common views, current situations, modern tactics, tangible emerging consequences, etc. The main objective here is to analyze the benefits of this study by investigating the past. Since this article analyzes what exists and is descriptive, the data is being retrieved by conducting a cross-sectional survey method about different features that are relevant by sampling the population. The main aim of this study is to explore the adoption of mobile banking technology by consumers. Based on the values of different variables such as affective commitment (AC), transaction convenience (TC), perceived ease of use (PEU), perceived reliability (PR), pre and post benefits (PPB), service, system, and information quality (SSIQ), bank trust (BT), and profitability (P), the inter-relationship between them and the adoption of m-banking technique by the users in banking technology. The model is investigated by examining the hypothesis and identifying the relationship that exists between these different parameters. A simple linear regression method is implemented using the Statistical Package for the Social Sciences (SPSS) software.

## Introduction

Nowadays, mobile technology plays a vital role in many industries such as banking on managing stocks and finances, business firms sell their products through applications and websites. Mobile banking services such as payment systems influence people's lives more dramatically than other innovations in the recent times (1). Mobile technology made banking services such as payment, transaction, and other services easy through the online mode instead of offline mode. Even the adoption of mobile banking services increased day-by-day by the consumers, still mobile payments do not become ordinary to many of the public (2). Due to the growth of mobile network technology, there is a need for security infrastructure for mobile payments. The security measures give trust to the consumers to adopt mobile banking at anytime from anywhere.

For achieving a successful outcome in business, mobile technology is recognized as competitive and creative equipment that provides better online transaction options (3). Recently, cellphones are being widely used as a tool by people for online shopping, banking, paying bills, etc. Our day-to-day routine lives have been changed through the adoption and development of mobile technology that modifies the way of communication, approaches for selling and buying goods and services, and also the mode of information collection. Such corresponding correlation arises in a constructive environment that has no limitation regarding the time or place (4).

The rapid development in mobile technology and the protection framework facilitate the people to perform m-banking transactions at any time from anywhere, whereas m-banking comprises financial service which is implemented directly or indirectly over a network of wireless telecommunication (3). In the financial sector, the services applied by mobile are referred to as mobile financial services (MFSs) they also include mobile banking and mobile payment. Several studies were conducted to examine the peoples' mindset in view of understanding the mode of adopting mobile banking as a unique and usual service (5). According to the statistics, the level of usage of smartphones has increased from 35 to 80% in 2020, also worldwide, the number of mobile phone beneficiaries is about 6.8 billion, though each mobile was fully activated with the internet. Thus, mobile banking helps in the development of bank access rates which influences bank perception (6). In the banking sector, it is observed that most of the customers have switched to smartphone, apps, or tablets which are highly utilized in their day-to-day life for shopping, entertainment, learning, socializing, and jobs, and these mobile-centric customers are preferred by more bankers (7). For example, a bank in Japan, Jibun Bank whose principal communication with customers is through the mobile channel and was the first bank that affords their complete banking services and products over mobile channels. With the help of cameras and mobile phone, customer has the facility to start a new account with their bank. This mode of banking enables the bank to improve their customer service over anywhere at any time basis. Also, the different mobile banking services are payment, web-related shopping, fund transfer, and web-related marketing. This Jibun Bank has reached more popular for its efficient services within the short duration of their mobile banking introduction (8). Through this, they have attained above 500,000 new bank accounts in their bank. In the initial days, the bank had the facility to send usual text messages related to money transfer and withdrawal and these services were updated to provide its customer a complete functional banking experience (9).

Mobile banking is adopted by many consumers as it is easy to open an account, pay bills, transfer funds, do mobile-based shopping, etc. Many banks in China and America who introduced mobile banking for all their services experienced high demand for their services through mobile banking (10). But many challenges were faced by these banks due to no face-to-face interactions. Trust is an important factor in mobile banking but lack of interaction led to the risk of uncertainty and anonymity. On the other hand, the consumers also suffer from uncertainty whether the bank is trustworthy are not.

During the last decades, mobile banking became relevant all over the world. The number of fund transfers through mobile banking also increased since last year, but mobile banking has many security problems. Sometimes, the security setting of mobile phones can be overridden by the virus in smartphones (11). Many banks open their banking applications, these apps should be updated frequently otherwise the consumers can be vulnerable to the attacks such as DDoS attacks, phishing, spoofing, corporate account takeover, and skimming (12).

The major contribution of the article is:

- To analyze the benefits of this study by investigating the past.
- To explore the adoption of mobile banking technology by consumers.

The remaining article is arranged accordingly. Section Literature Review presents the related work and the hypothesis is formulated in Section Hypothesis Development and Research Methodology. The results in terms of linear regression and descriptive analysis are provided in Section Results and the article is concluded in Section Conclusion and Discussions.

## Literature Review

In the modern world, many firms use systems of mobile banking to communicate with the customers. The payment system in mobile banking services benefited people's lives. This article aims to analyze the effect of people using the technology of mobile to make banking services more accessible. Literature on mobile banking adoption, electronic banking services, mobile payment service user acceptance are reviewed.

Hamidi et al. (13) studied the influence of mobile banking adoption on consumer engagement and satisfaction utilizing the customer relationship management (CRM) system, which is the most essential aspect in banking industry. CRM is also seen as a critical role for enhancing client satisfaction in mobile banking. The statistical study performed evaluated the dialogue between the bank's customer sector and their client. The statistical analysis findings have a favorable influence on consumer interactions and satisfaction.

Geebren et al. (14) investigated the importance of consumer satisfaction in mobile eco-systems that used electronic banking services, particularly in developing nations. This entailed researching consumer satisfaction in mobile banking, with a focus on the importance of trust. To determine consumer satisfaction, structural modeling using partial least squares (PLS–SEM) methods were employed to examine the data, and trust demonstrated that customer contentment had a beneficial influence.

The relevance of an early trust theoretical model in mobile payment service user acceptance was highlighted by Gao et al. (15). The initial trust theoretical model highlighted the facilitators and barriers to user trust in m-payment services. The links in the original trust theoretical model were assessed using partial least squares structural modeling (PLS–SEM). The findings may be used in m-payment adoption research and practice in a variety of ways. In total, 52.3% of the difference in usage intention was explained by the current model.

Hentzen JK et al. (16) offered a mobile technology that allows for vital involvement, as well as an explanation of how a retirement app might assist people in planning for their post-retirement strategy. The available literature survey data were used to evaluate a sample of 440 Australian pension fund members. The findings show that consumers' perceived financial security, financial self-efficacy, retirement planning involvement, future consequences consideration, and perceived usefulness with a mobile retirement app have direct and indirect effects on their expected engagement through their goal to adopt the app.

Zhu et al. (17) investigated existing technology designs, including mobile banking, used by rural communities in six Chinese regions. According to the findings, interpersonal and mass communication channels have a bigger influence than organizational communication channels. Mobile banking should be examined since it can assist alleviate the lack of access to financial goods and financial infrastructure in rural areas.

Afeti et al. (18) developed a mobile payment technology for payment for micro-businesses. The study draws on the transaction cost theory and the task-technology fit (TTF) theory as the assumed lens. In total, 20 micro-businesses based on qualitative data were analyzed and the research findings denote those micro-businesses adoption of mobile payments results in strategic and operational benefits.

Alamoudi et al. (19) proposed that the mobile technology acceptance lookalike was changed as we investigated consumers' acceptance of mobile shopping in general stores by examining transaction convenience, usefulness, attitudes, ease of use, transaction speed, optimism, and personal innovativeness. A total of 351 respondents completed the questionnaire evaluation. Consumers are willing to use mobile shopping channels if the system is clear and straightforward to use.

Jebarajakirthy et al. (20) used a comprehensive moderated mediation framework to evaluate the influence of online convenience aspects on mobile banking uptake. Covariance-based structural equation modeling and the process macro are utilized to test these predictions. This study examines how convenience characteristics influence mobile banking adoption intentions.

Abdinoor et al. (21) studied the adoption of mobile financial services in Tanzania with the use of a technology acceptance model. To select the sample from data collection, a random sampling technique was used. The user and non-user of mobile financial services were included in the sample. Zhang et al. (3) investigated customers' use of mobile technology to help them with banking services and activities, as well as the variables that impact their adoption and engagement. Here, the analysis is done by the structural equation modeling technique to know the consumers' intentions toward mobile banking. The result examines the adoption of mobile banking apps to facilitate bank consumers' banking services.

Tiwart et al. (22) researched the variable that influences adoption in Commercial Bank Ethiopia. The author used factors such as perceived ease of use (PEOU), infrastructure (INF), security (SEC), trust, and e-banking adoption. Here, a structural equation model based on least partial square analysis is used. The result of this analysis proves that the trust of customers mediates between PEOU, INF, SEC, and e-banking adoption and realized the factors that guess the purpose and adoption of e-banking by Ethiopian customers.

The problem identified in mobile banking services is critical financial services through mobile banking because it has security problems.

## Hypothesis Development and Research Methodology

### Participant's Demographic Information

This study is mainly conducted in China by taking the top e-banks that utilize m-banking. The questionnaire is distributed to both the users and staff of the bank. Within 6 months, 293 questionnaires were completed. These details were taken into consideration in this study. The details of the questionnaires were obtained online and also provided to the users on their official bank pages. The information obtained in the questionnaire is provided in Tables 1, 2.

**Table 1:** Demographic analysis.

**Demographics variables**	**Percentage**	**Number**
**Sex**		
Female	54.50	121
Male	45.49	101
**Age**		
1–20	3	25
20–30	59	120
30–40	15	14
>40	23	63
Education		
Master of science	12	25
Bachelor of science	15	32
Engineering	20	57
Medicine	18	60
Diploma	40	85
Associate degree	17	14
PhD	5	7

**Table 2:** Participant details.

**Bank types**	**User rating**
State-owned	3.25
Joint-stock	2.14
Postal savings	3.25
Agricultural bank	2.52
Commercial city bank	1.54

### Participant Details

The information of the customers is mainly obtained based on their m-bank usage. The five main banks such as state-owned, joint-stock, postal savings, commercial city bank, and the agricultural bank were selected. Each participant was asked to say about their experience of their usage.

**Figure 1:**
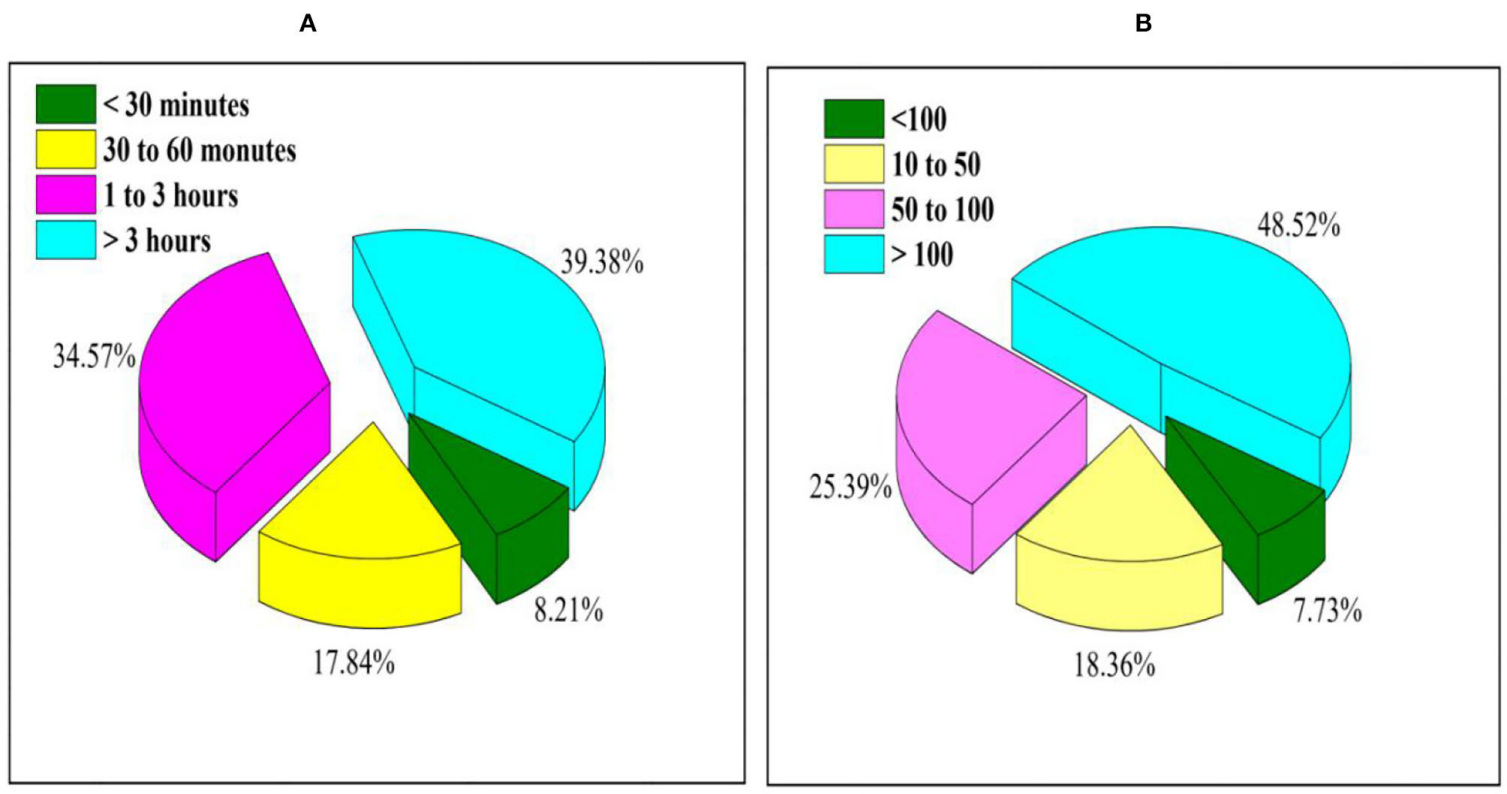
Usage analysis of m-banking application **(A)** Users using m-banking during the day and **(B)** users' friends and references using mobile banking.

The amount of time spent by each user on average is provided in Figure 1A. As seen in Figure 1A, more than 39.38% of users are using more than 3 h a day and a minimum of is online Conceptual Model for less than 1 h. In total, 8.21% of the users only use the application for a limited amount of time (below 30 min).

Figure 1B presents the percentage of people the users have referred for mobile banking. Based on Figure 1B, a total of 48.52% has more than 100 contacts who have been referred to the m-banking. The increase in m-banking contact can improve efficiency.

### Conceptual Model

**Figure 2:**
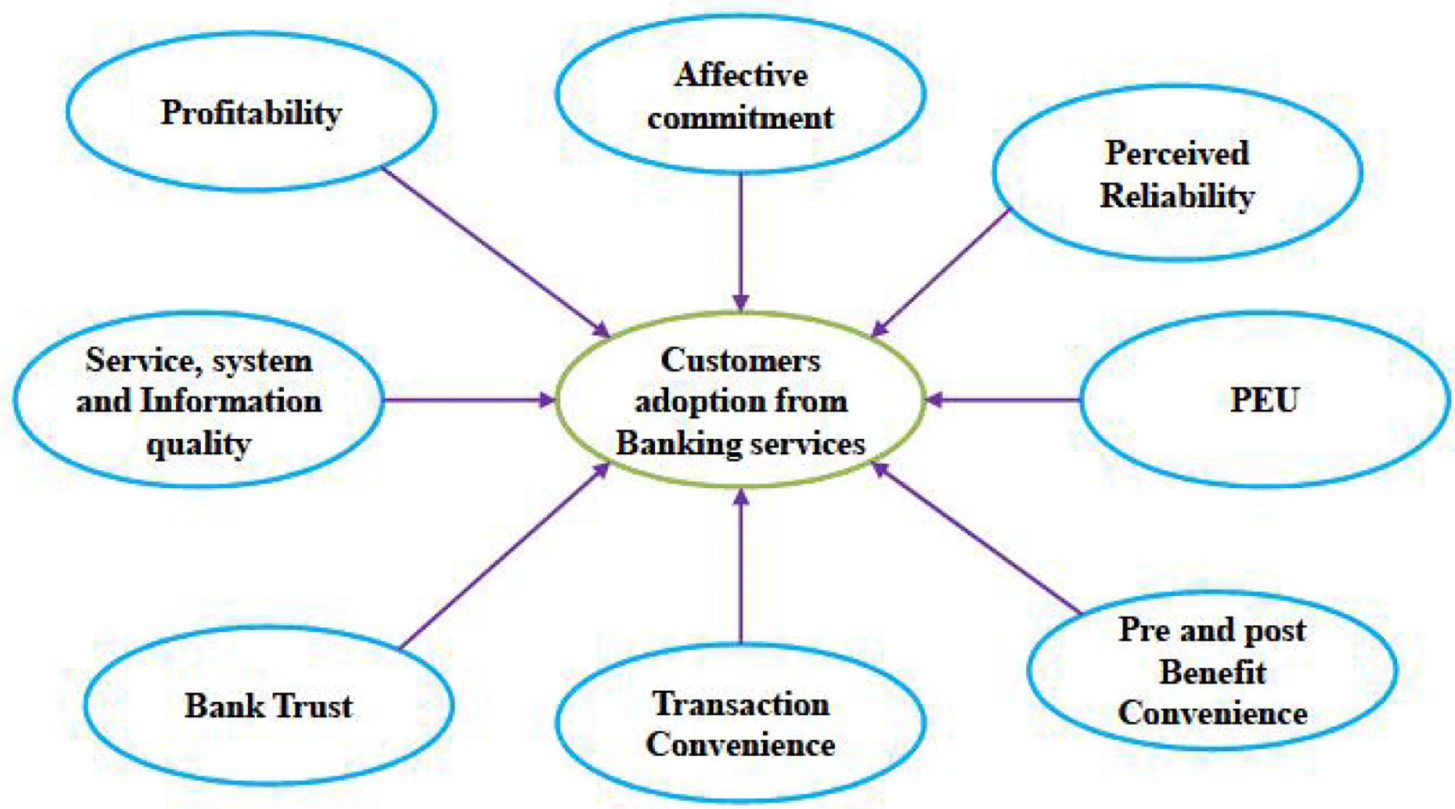
Conceptual modeling structure.

The main aim of this research is to identify the usage of mobile technology of consumers for the banking system. The hypothesis provided for each variable is explained in detail in this section and the conceptual model is provided in Figure 2. The measurement instrument used for this study is presented in Table 3.

**Table 3:** Measurement instrument to analyze different parameters.

**Variables**	**Measurement instrument**
Bank trust	The bank helps me to use the mobile device of the consumer for accessing the services BT1: The m-banking is trustworthy BT2: The m-banking mainly satisfies the promises and commitments
Pre and post benefit convenience	The m-banking resolves the problems in a fast manner Obtaining the follow-up service is easy The solutions provided by m-banking is fast and reliable The banking service is provided with little effort The services are easier to avail The problem is resolved fastly The time taken by it is reasonable
Service, system, and information quality (SSIQ)	Convenient access of m-banking services Easy navigation of m-banking services Visual attractiveness of m-banking services Accurate information of m-banking services Up-to-date information of m-banking services Dependable services provided Personalized m-banking services Professional m-banking services Timely m-banking services
Transaction convenience	Easier to complete the transaction Fast service access Little effort to complete the transactions
Perceived ease of use	Usability of mobile devices for banking Interaction with the mobile devices for banking Skillset achieved in accessing mobile devices for banking
Perceived reliability	To select the supporting technology for mobile banking Ensuring that the technology supporting m-banking does not fail The technology used is reliable
Portability	Increased customer interaction Increased customer visit

### Hypothesis Modeling

#### Service, System, and Information Quality (SSIQ)"

System quality mainly measures the efficiency of the overall m-banking system in terms of service provider anonymity. The system quality of the m-banking mainly depends upon the ease of use, the attractiveness of the application, trust of the user, etc. If the user trusts the system quality, then they mainly select the system for future transactions. Service Quality mainly relies on the service provided by the m-banking system and it also evaluated the effectiveness of the service in terms of personalization, reliability, delay, etc. Hence, service quality is crucial to improve the quality of m-banking since poor-service quality can result in minimal user trust and satisfaction. Information quality mainly implies that high-quality information is reliable, complete, accurate, relevant, and accessible. The information quality is an important variable that improves the usage, trust, and satisfaction of the customers in the m-banking system.

**Hypothesis (H6):** The service, system, and information quality had a positive relationship in improving the m-banking quality and user satisfaction.

#### Affective Commitment

Affective obligations between business partners reflect mental reliance on others and are founded on emotions, loyalty, and dependency. The psychological bond in this example demonstrates an emotional commitment. Support for differences in particular data among new and long-term clients. Based on their differing expectations, new and loyal clients behave in the same way.

**Hypothesis (H1):** The interaction of customers helps to develop a good relationship with the affective commitment of a person to the bank which is focused on interaction.

#### Pre- and Post-benefit Convenience

The time and attempt to acquire some benefits of services is said to be benefit convenience. Some of the components of benefit convenience are fast service, timely services, and bank employee attitudes. Sometimes in banks, the consumer needs to visit repeatedly to avail specific service. But in m-banking, consumers can avail any services on the go from the home itself. So, the time and attempt to acquire the service became very easy. Therefore, benefit convenience shows a positive result in mobile banking adoption (23). The time and attempt to contact a service provider for a particular service is said to be post benefit convenience. In m-banking, the service provider can be contacted and solve the grievances within a few clicks through various means such as email, live chat, and toll-free numbers. But in offline banking, the consumer needs to visit the branch and wait for the service provider to solve the problem in service. So, the post benefit convenience shows a positive result in mobile banking adoption (20).

**Hypothesis (H5):** The pre- and post-benefit convenience are positively interrelated with m-banking.

#### Transaction Convenience

The fast and simple way to complete the transaction is said to be transaction convenience. Acquiring the service of transaction in a lesser time is the main component of transaction convenience. Other components of transaction convenience are uninterrupted transaction, transaction confirmation, easy checkout, and price inconsistency. By using m-banking, the transaction can be done in any place at any time in a few clicks and can do several transactions simultaneously. But in offline banking, consumer needs to wait in a queue for availing a transaction service. Hence, transaction convenience shows a positive result in mobile banking adoption (24).

**Hypothesis (H2):** The transaction convenience is positively interrelated with m-banking.

#### Profitability

Customer participation in an organization's mobile banking may increase customer value and profitability (25).

**Hypothesis (H8):** Profitability shows positive results in mobile banking adoption.

#### Perceived Reliability

Perceived reliability is specified as the limit to which the individual has independently considered the technology as a faithful one. In order to offer a trustworthy technical service, the system reliability is important that enables the user to attain the aimed objective. An undependable technology that is employed by an individual seems to be low confidence and such technology utilization should be restricted (26). Research related to mobile technology and information system has revealed that a technology with perceived reliability possesses has an important impact on customers' perceived satisfaction, detected value, and observed quality. Also, customers' understanding level of trust is examined and analyzed as an antecedent of confidence in an organization through the mobile commerce setting. Though mobile is a wireless technology, it is easily susceptible to violations or attacks (27). Mostly, the customers were insisted to share their personal data while consuming and shopping services performed through mobile technology such as date of birth, debit and credit card details, funds, and address. The reliability of mobile technology should be improved thereby increasing customers' belief in securing their personal data. The postulated hypotheses are mentioned as follows:

**Hypothesis (H4):** Perceived reliability in m-banking helps to improve the consumer's trust and ease of use of banking services.

#### Banking Trust

In this hypothesis, trust is considered as a faith of competence, integrity, ability, and benevolence that the individual has toward each other. Trust has become an important reducing feature of risk, whereas the e-commerce connection is considered as a naturally risky factor (28). Also, trust in banking is specified that it is linked to reliability and perceived privacy. Generally, the primary interaction between the customer and banker will significantly affect the trust progress in them.

**Hypothesis (H7):** Based on their usage of mobile devices and attitude, the consumer trust toward M-banking increases.

#### Perceived Ease of Use (PEU)

Perceived ease of use evaluates the increase in the amount of work required to acquire a job utilizing new technologies on an individual basis. In the effort condition, the simplicity of use of mobile innovation is critical for including customers in the co-creation of value experiences (29). Mobile technology's ability to increase advantages for bank clients has been widely shown, and its simplicity of use is a key factor in consumer acceptability. PEU has been linked to advanced tackling new technologies in the banking industry in several research. However, the launch was difficult, and it was claimed that customers' inclination to utilize mobile banking services is not intrinsically related to employment prospects. As a consequence, it indicates that study into PEU and its influence on consumer attitudes, as well as the interactive method, are both worthwhile endeavors (30–32).

**Hypothesis (H3):** Customers' views about the usage of mobile devices for facilitating financial services are favorably associated with their perceived ease of use (PEU).

## Results

### Scale Validity and Reliability

The reliability of the scales was assessed using Cronbach's alpha values. Goodness-of-fit approaches were used to assess the overall model fit for confirmatory factor analysis (CFA). The RMSEA was 0.09, the comparative fit index (CFI) was 0.99, the goodness-of-fit (GFI) was 0.98, and the standardized root mean square residual (RMR) was 0.06. The X2 to df ratio was 4.14, the root mean square error of approximation (RMSEA) was 0.09, the CFI was 0.99, the GFI was 0.98, and the standardized RMR was 0.06. As indicated in Table 4, the reliability coefficients of all constructs were more than 0.87, which is higher than the 0.9 thresholds. The extracted average variance was used to assess convergent validity (AVE). Because the AVE values ranged from 0.58 to 0.92, convergent validity was not an issue. The AVE scores were lower than the squared correlations between pairs of constructs, indicating discriminant validity in the data set. Table 4 shows the results of the Cronbach's alpha and AVE tests.

**Table 4:** Hypothesis testing.

**Variables**	**Cronbach's alpha**	**Average variance extracted** **(AVE)**	**AC**	**TC**	**PEU**	**PR**	**PPB**	**SSIQ**	**BT**	**P**
Affective commitment (AC)	0.78	0.58	0.77							
Transaction convenience (TC)	0.96	0.87	0.55	0.94						
Perceived ease of use (PEU)	0.97	0.88	0.62	0.68	0.95					
Perceived reliability (PR)	0.98	0.91	0.51	0.52	0.56	0.96				
Pre and post benefits (PPB)	0.98	0.92	0.55	0.74	0.66	0.53	0.97			
Service, system, and information quality (SSIQ)	0.95	0.82	0.47	0.56	0.66	0.44	0.74	0.92		
Bank trust (BT)	0.87	0.68	0.72	0.46	0.48	0.52	0.49	0.42	0.83	
Profitability (P)	0.83	0.64	0.67	0.42	0.44	0.50	0.46	0.39	0.80	0.84

### Linear Regression Test

Regression tests are run when the allowable alpha coefficient for the scales has been determined. Table 5 shows the regression test results for each of the assumptions discussed in the following sections. Affective commitment (AC) and interaction were investigated, and the correlation coefficient 0.297 and likelihood of significance 0.000 indicate that there is a positive and significant relationship between the two. The regression test supports this positive relationship, showing that the interaction variable may be able to predict AC variation.

**Table 5:** The results of the linear regression test.

**The dependent variable**	**Independent variable**	**Number theory**	**Test significance *P*-value**	**The correlation coefficient R^**2**^**	**Result**
Affective commitment (AC)	Interaction	H1	0.000	0.297	Accepted
Transaction Convenience (TC)	Interaction	H2	0.000	0.339	Accepted
Perceived ease of use (PEU)	Satisfaction	H3	0.000	0.333	Accepted
Perceived reliability (PR)	Interaction	H4	0.000	0.172	Accepted
Pre and post benefits (PPB)	Interaction	H5	0.000	0.242	Accepted
Service, system, and information quality (SSIQ)	Satisfaction	H6	0.000	0.430	Accepted
Bank trust (BT)	Interaction	H7	0.000	0.347	Accepted
Profitability (P)	Interaction	H8	0.000	0.230	Accepted

With a correlation value of 0.333 and a probability of significance of 0.000, the following test demonstrated a positive relationship between perceived ease of use and satisfaction (PEU). The correlation coefficient between the variables perceived reliability (PR) and interaction was 0.172, with a probability of significance of 0.000, demonstrating a positive link between the two variables. The correlation value of 0.430 and the probability of significance of 0.000 indicated a positive relationship between satisfaction and service, system, and information quality (SSIQ) (3).

### Descriptive Data

The items in this questionnaire were answered using a five-point Likert scale, with the alternatives being: very low (1), low (2), medium (3), high (4), and very high (5). Table 6 shows the SD, middle value (MV), and lowest value (LV) of the descriptive data received from the surveys (3).

**Table 6:** Descriptive data analysis.

**Variables**	**Standard deviation (SD)**	**Middle**	**Highest**	**Lowest**
Affective commitment (AC)	1.208	2.52	5	1
Transaction convenience (TC)	1.081	3.36	5	1
Perceived ease of use (PEU)	1.061	3.21	5	1
Perceived reliability (PR)	1.689	2.92	5	1
Pre and post benefits (PPB)	1.393	2.67	5	1
Service, system, and information quality (SSIQ)	1.206	2.79	5	1
Bank trust (BT)	0.955	3.31	5	1
Profitability (P)	1.220	3.10	5	1

## Conclusion and Discussions

Mobile banking is a cost-effective way to reach customers. The different activities that can be performed by the consumers in m-banking are checking their bank balance, making transactions, making investments, getting account statements, paying bills, etc. The motivation for user adoption and use of mobile banking is examined in this research. Positive connections between profitability, trust, and transaction convenience to utilize mobile banking have been found in previous studies. Users' confidence in using mobile banking services, enjoyment, utility of the system, etc., are not the same thing. The participants were unconcerned about the danger of fraud, system dependability, or perceived privacy while building and extending faith in their banks and mobile services. Because of today's technology-driven lifestyle, individuals are receptive to embracing new technologies that are compatible with their mobile phones.

To add value to practice, we must obtain convincing and widespread results from both users and mobile banking service providers. Like mobile phones, smartwatches have become more popular among people nowadays. With this wearable technology, banks have the greatest responsibility to fulfill hedonic and social needs. The purpose and use of this research are common to all consumers irrespective of various countries, populations, and socio-economic status. The variables used in this work are, namely, affective commitment (AC), transaction convenience (TC), perceived ease of use (PEU), perceived reliability (PR), pre- and post benefits (PPBs) service, system, and information quality (SSIQ), bank trust (BT), and profitability (P). Consumer behavior such as untrustworthiness, fraudulence, and so on, as well as mobile banking transaction risks such as financial, privacy, and cyber security, are important risk concerns that may be addressed in future research developments.

The problems and risks that arise by the consumers and banking sector for adopting mobile banking services would be reduced by the scientific researchers using various technologies and methodologies in the future. The results in this article show the different statistical surveys and believed that it is true for both mobile banking consumers and non-mobile banking consumers with various socioeconomic environments. But when we consider globally each country had its own rules and regulations regarding mobile banking transactions, services, and products. And these rules and regulations react differently in different countries.

## Data Availability Statement

The original contributions presented in the study are included in the article/supplementary material, further inquiries can be directed to the corresponding author/s.

## Author Contributions

Both authors listed have made a substantial, direct, and intellectual contribution to the work and approved it for publication.

## Conflict of Interest

The authors declare that the research was conducted in the absence of any commercial or financial relationships that could be construed as a potential conflict of interest.

## Publisher's Note

All claims expressed in this article are solely those of the authors and do not necessarily represent those of their affiliated organizations, or those of the publisher, the editors and the reviewers. Any product that may be evaluated in this article, or claim that may be made by its manufacturer, is not guaranteed or endorsed by the publisher.

## References

[1] Patil P Tamilmani K Rana NP Raghavan V. Understanding consumer adoption of mobile payment in India: Extending Meta-UTAUT model with personal innovativeness, anxiety, trust, and grievance redressal. Int J Inf Manage. (2020) 54:102144. 10.1016/j.ijinfomgt.2020.102144

[2] Martin J. 7 reasons mobile payments still aren't mainstream. (2016).

[3] Zhang T Lu C Kizildag M. Banking “on-the-go”: examining consumers' adoption of mobile banking services. Int J Quality Serv Sci. (2018) 10:279–95. 10.1108/IJQSS-07-2017-0067

[4] Sharma SK Al-Muharrami S. Mobile banking adoption: key challenges and opportunities and implications for a developing country. In: Emerging Markets from a Multidisciplinary Perspective. Springer, Cham. (2018). p. 75–86. 10.1007/978-3-319-75013-2_7

[5] Riquelme HE Rios RE. The moderating effect of gender in the adoption of mobile banking. Int J Bank Market. (2010) 28:328–41. 10.1108/02652321011064872

[6] Sanakulov N Karjaluoto H. Consumer adoption of mobile technologies: a literature review. Int J Mobile Commun. (2015) 13:244–75. 10.1504/IJMC.2015.069120

[7] Shareef MA Baabdullah A Dutta S Kumar V Dwivedi YK. Consumer adoption of mobile banking services: An empirical examination of factors according to adoption stages. J Retail Consumer Serv. (2018) 43:54–67. 10.1016/j.jretconser.2018.03.003

[8] Hanafizadeh P Behboudi M Koshksaray AA Tabar MJS. Mobile-banking adoption by Iranian bank clients. Telemat Inform. (2014) 31:62–78. 10.1016/j.tele.2012.11.001

[9] Srivastava M Fernandes S. An empirical study to measure consumer adoption of mobile banking services. Int J Public Sector Perform Manage. (2022) 9:165–89. 10.1504/IJPSPM.2022.119834

[10] Narteh B Yeboah-Asiamah E Mackin EA. Analysis of young banked and unbanked customers' usage, satisfaction, trust and loyalty for mobile money services in Ghana. Int J Bus Syst Res. (2022) 16:40–64. 10.1504/IJBSR.2022.119602

[11] Nur T Panggabean RR. Factors influencing the adoption of mobile payment method among generation Z: the extended UTAUT approach. J Account Res Organiz Econ. (2021) 4:14–28. 10.24815/jaroe.v4i1.19644

[12] Muhal MA Luo X Mahmood Z Ullah A. Physical unclonable function based authentication scheme for smart devices in Internet of Things. In: 2018 IEEE International Conference on Smart Internet of Things (SmartIoT). (2018) p. 160–165. IEEE. 10.1109/SmartIoT.2018.00037 32749985

[13] Hamidi H Safareeyeh M. A model to analyze the effect of mobile banking adoption on customer interaction and satisfaction: a case study of m-banking in Iran. Telemat Inform. (2019) 38:166–81. 10.1016/j.tele.2018.09.008

[14] Geebren A Jabbar A Luo M. Examining the role of consumer satisfaction within mobile eco-systems: Evidence from mobile banking services. Comput Human Behav. (2021) 114:106584. 10.1016/j.chb.2020.106584

[15] Gao L Waechter KA. Examining the role of initial trust in user adoption of mobile payment services: an empirical investigation. Inf Syst Front. (2017) 19:525–48. 10.1007/s10796-015-9611-0

[16] Hentzen JK Hoffmann AO Dolan RM. Which consumers are more likely to adopt a retirement app and how does it explain mobile technology-enabled retirement engagement? Int J Consumer Stud. (2021) 46:368–90. 10.1111/ijcs.12685

[17] Zhu Q Lyu Z Long Y Wachenheim CJ. Adoption of mobile banking in rural China: Impact of information dissemination channel. Socio-Econ Plann Sci. (2021) 101011. 10.1016/j.seps.2021.101011

[18] Afeti EY Owusu A. Impact of mobile payments on micro-business activities: a developing country experience. In: Digital Innovations, Business and Society in Africa. Springer, Cham. (2022). p. 75–95. 10.1007/978-3-030-77987-0_4

[19] Alamoudi H. Examining Retailing Sustainability in the QR Code-Enabled Mobile Payments Context During the COVID-19 Pandemic. Int J Customer Relationship Market Manage. (2022) 13:1–22. 10.4018/IJCRMM.289210

[20] Jebarajakirthy C Shankar A. Impact of online convenience on mobile banking adoption intention: a moderated mediation approach. J Retail Consumer Serv. (2021) 58:102323. 10.1016/j.jretconser.2020.102323

[21] Abdinoor A Mbamba UO. Factors influencing consumers' adoption of mobile financial services in Tanzania. Cogent Busin Manage. (2017) 4:1392273. 10.1080/23311975.2017.1392273

[22] Tiwari P. Electronic banking adoption in Ethiopia: an empirical investigation. SN Bus Econ. (2021) 1:1–28. 10.1007/s43546-021-00114-0

[23] Shen YC Huang CY Chu CH Hsu CT. A benefit–cost perspective of the consumer adoption of the mobile banking system. Behav Inf Technol. (2010) 29:497–511. 10.1080/01449290903490658

[24] Trialih R Astuti ES Azizah DF Mursityo YT Saputro MD Aprilian YA. How mobile banking service quality influence customer satisfaction of generation x and y? In: 2018 International Conference on Information and Communication Technology Convergence (ICTC). (2018) p. 827–32. IEEE. 10.1109/ICTC.2018.8539720

[25] Gupta SD Raychaudhuri A Haldar SK. Information technology and profitability: evidence from Indian banking sector. Int J Emerg Market. (2018) 13:1070–87. 10.1108/IJoEM-06-2017-0211

[26] Prabhu Kavin B Ganapathy S. EC(DH)2: an effective secured data storage mechanism for cloud based IoT applications using elliptic curve and Diffie-Hellman. Int J Internet Technol Secur Transact. (2020) 10:601–17. 10.1504/IJITST.2020.10029366

[27] Prabhu Kavin B Ganapathy S. A new digital signature algorithm for ensuring the data integrity in cloud using elliptic curves. Int Arab J Inf Technol. (2021) 18:180–90. 10.34028/iajit/18/2/6

[28] Prabakaran S Ramar R Hussain I Kavin BP Alshamrani SS AlGhamdi AS. Predicting attack pattern via machine learning by exploiting stateful firewall as virtual network function in an SDN network. Sensors. (2022) 22:709. 10.3390/s22030709 35161456 PMC8839531

[29] Ramachandran V Ramalakshmi R Kavin BP Hussain I Almaliki AH Almaliki AA. Exploiting IoT and its enabled technologies for irrigation needs in agriculture. Water. (2022) 14:719. 10.3390/w14050719

[30] Adam MNM Adam MNM Idris AAA Ali ASM Khalid IO Evaluation Evaluation of the effect of time of fixation and microwave treatment on quality of fatty tissue fixation in breast cancer specimens. SPR. (2021) 2:445–51. 10.52152/spr/2021.166

[31] Mshvidobadze T. Bioinformatics as emerging tool and pipeline frameworks. SPR. (2021) 2:361–5. 10.52152/spr2021.156

[32] Dani R Juyal D Rawal YS. A Critical analysis of the restaurant industry's effect on environment sustainability. SPR. (2021) 2:385–92. 10.52152/spr/2021.159

